# The Current from the Main

**Published:** 1902-11-01

**Authors:** 


### THE CURRENT FROM THE MAIN.

### I.

The introduction of the electric light into houses and institutions has led to numerous attempts to utilise the current in place of the troublesome primary batteries which are so constantly getting out of order and breaking down, but it is often difficult to do this without danger, and to make it serve all purposes. If we have a continuous current to start with it is possible to obtain any variety of current by suitable apparatus, though it may not be always cheaper or easier to do so than to use primary batteries. Both in this case and where the current from the main is an alternating one the greatest care must be taken to keep the patient who is under treatment safely insulated from the earth, otherwise severe and even fatal shocks may be given by so-called earth currents. To make this plain, if he is placed for treatment in a circuit which is sufficiently controlled by a resistance before its entrance into his body, no other resistance or barrier is put into the wire on the side of exit. But should his feet be on a wet floor, connected, perhaps, by iron joists in the building with the earth, and there be any other wire from the main connected with the earth here, accidentally or otherwise, a powerful current may pass from this other wire through the patient and what we may call the unguarded wire of exit from his body. When large currents are sent into an institution, or the earth is used for the return current of the main, such a shock may be easily fatal. Thus if the waste pipe from an electric bath is not cut off, or even if a continuous stream of water pass from the bath while in use to the drains, an earth current of this kind may arise. Again, a nurse standing on a wet floor by a bath and putting a hand into it might conceivably form a circuit between the earth and the return wire of the bath current. However, with reasonable care it is easy to prevent any danger of this kind. When we have to do with an alternating current it is not possible (without an expensive dynamo) to use it instead of a continuous current battery for many purposes. We cannot develop Roentgen rays directly from it, nor obtain the continuous current for the treatment of paralysed limbs. But it is found that this alternating or sinusoidal current is practically as good as a continuous one for most therapeutic purposes, and can be used instead of it; it is not so sedative to pain, but its effect on the nutrition of muscles is probably just as valuable. For diagnosis and muscle testing by comparing the Faradic and Galvanic reactions, we still require a constant current battery, though it is easy enough to work the Faradic coil from the sinusoidal main by interposing a lamp resistance; but for actual treatment the sinusoidal current itself is far easier and cheaper to work with. It is always ready, rarely out of order, and one person can attend to and treat several patients at once. Where there are a large number of cases of wasted limbs from lead poisoning, infantile and crutch or splint paralyses to be restored to health as quickly as possible, this is an enormous gain. Instead of the attendant working at a single patient for ten or fifteen minutes with a primary battery which must be put aside to rest after a time, the patients seat themselves at various arm and foot baths, and suitable currents are turned on. With children, too, there is much less fright and screaming. After the first few days many of them look forward to their baths, whether general or local, and sit very happily by their elders playing with toys. The current when applied directly to a limb by an electrode is disagreeable, and therefore it is generally applied through water. If the trouble is strictly limited to the forearm or leg, that limb only is immersed in the bath; but if it is nearer the shoulder or hips the arm must be placed in one pan and the other arm or foot in a second, so that the current may be sent through the injured part from one pan to the other; or a large well covered electrode is held in the hands or placed on the nape of the neck instead of the second pan. In St. Bartholomew's Hospital they have besides arm baths a German invention consisting of a chair with a trough for each arm and two pans for the feet of the patient, so that currents can be sent through two or more limbs and the trunk in the easiest manner. Finally the sinusoidal current applied by a general bath to the whole body is probably the most efficacious treatment we possess for rheumatoid arthritis and certain forms of neurasthenia. The current here must be even more carefully controlled than for local applications. A patient lying in a long bath is quite helpless, and serious pain and fright at the least may follow any carelessness. A trustworthy resistance and rheostat are necessary, the current must not be turned on or off suddenly, and the attendant should always test the current in the bath by his hands held far apart before the patient gets in. It is desirable to insert a milliampere meter into the circuit to show the current actually passing through the patient, but with the precautions mentioned that is not absolutely necessary in all cases.

